# Supplementary material for: The evolution of antibiotic resistance islands occurs within the framework of plasmid lineages
Source: Nat Commun. 2024 May 29;15:4555. doi: 10.1038/s41467-024-48352-8 (PMC11137137; doi:10.1038/s41467-024-48352-8)
Supplement: Supplementary file 3 — Description of Additional Supplementary Files [file 41467_2024_48352_MOESM3_ESM.pdf]

<u>Description of Additional Supplementary Files</u>

Supplementary Data S1: A list of 9,225 replicons used in searching for coARGs.

Supplementary Data S2: ARG families in plasmid resistance islands (REI). The table is ordered according to the frequency in REI.

Supplementary Data S3: The frequency of significant cooccurring ARGs (coARGs) in replicons, syntenic blocks and transposable elements.

Supplementary Data S4: Biased distribution of transposases and site-specific recombinases (SSRs) in plasmids and chromosomes.

Significant occurrence of SSRs in plasmids or chromosomes was tested using two-sided Fisher's exact test (α=0.05) corrected with false discovery rate (FDR). The table is ordered according to the frequency in REI.

Supplementary Data S5: A list of significantly co-occurring ARGs in ARG-encoding plasmids.

The table comprises three components: K: Klebsiella plasmids; E: Escherichia plasmids; S: Salmonella plasmids. Significant cooccurrence of ARG pairs from different gene families was tested using two-sided Fisher's exact test in ARGcarrying plasmids (α=0.05 and correction for multiple comparisons with FDR).

Supplementary Data S6: CSBs distribute biased towards specific PTU.
CSBs are shown in Figure 6.

Supplementary Data S7: Example pieces of resistance island.

CSBs are shown in Supplementary Data S7.
